# Sorbitol Destroyed Intestinal Microfold Cells (M Cells) Development through Inhibition of PDE4-Mediated RANKL Expression

**DOI:** 10.1155/2024/7524314

**Published:** 2024-05-02

**Authors:** Li Xiang, Wenxu Pan, Huan Chen, Wenjun Du, Shuping Xie, Xinhua Liang, Fangying Yang, Rongwei Niu, Canxin Huang, Minan Luo, Yuxin Xu, Lanlan Geng, Sitang Gong, Wanfu Xu, Junhong Zhao

**Affiliations:** ^1^Guangzhou Institute of Pediatrics, Guangzhou Women and Children's Medical Center, Guangzhou Medical University, Guangzhou 510623, China; ^2^Department of Gastroenterology, Guangzhou Women and Children's Medical Center, Guangzhou Medical University, Guangzhou, China; ^3^The First Affiliated Hospital of Jinan University, Jinan University, Guangzhou, China; ^4^The Second Clinical Medical School, Guangzhou Medical University, Guangzhou, China; ^5^The School of Pediatrics, Guangzhou Medical University, Guangzhou, China

## Abstract

**Objective:**

Microfold cells (M cells) are specific intestinal epithelial cells for monitoring and transcytosis of antigens, microorganisms, and pathogens in the intestine. However, the mechanism for M-cell development remained elusive.

**Materials and Methods:**

Real-time polymerase chain reaction, immunofluorescence, and western blotting were performed to analyze the effect of sorbitol-regulated M-cell differentiation *in vivo* and *in vitro*, and luciferase and chromatin Immunoprecipitation were used to reveal the mechanism through which sorbitol-modulated M-cell differentiation.

**Results:**

Herein, in comparison to the mannitol group (control group), we found that intestinal M-cell development was inhibited in response to sorbitol treatment as evidenced by impaired enteroids accompanying with decreased early differentiation marker Annexin 5, Marcksl1, Spib, sox8, and mature M-cell marker glycoprotein 2 expression, which was attributed to downregulation of receptor activator of nuclear factor kappa-В ligand (RANKL) expression *in vivo* and *in vitro*. Mechanically, in the M-cell model, sorbitol stimulation caused a significant upregulation of phosphodiesterase 4 (PDE4) phosphorylation, leading to decreased protein kinase A (PKA)/cAMP-response element binding protein (CREB) activation, which further resulted in CREB retention in cytosolic and attenuated CREB binds to RANKL promoter to inhibit RANKL expression. Interestingly, endogenous PKA interacted with CREB, and this interaction was destroyed by sorbitol stimulation. Most importantly, inhibition of PDE4 by dipyridamole could rescue the inhibitory effect of sorbitol on intestinal enteroids and M-cell differentiation and mature *in vivo* and *in vitro*.

**Conclusion:**

These findings suggested that sorbitol suppressed intestinal enteroids and M-cell differentiation and matured through PDE4-mediated RANKL expression; targeting to inhibit PDE4 was sufficient to induce M-cell development.

## 1. Introduction

Intestinal microfold (M) cells, playing a critical role in intestinal immunity, were a unique subset of intestinal epithelial cells (IECs) in the peyer's patches, which allowed immune responses to occur in response to intestinal pathogens/antigens through sampling antigens/pathogens from the luminal surface to the subepithelium, gaining access to lamina propria for the purpose of infection and propagation and dissemination, such as murine norovirus and reovirus [1, 2], mammalian orthoreovirus [3], *Salmonella typhimurium* (*S. typhimurium*) [4, 5] and *Candida albicans* (*C. albicans*) [6, 7]. Moreover, M-cell deficient in *Spib*^−/−^ mice led to develop chronic and severe colitis with increased bacterial dissemination and attenuated commensal-specific Th1 and Th17 responses [8], suggesting the contribution of intestinal M cells to maintain intestinal hemostasis under physiological conditions. Despite the critical role of M cells in intestinal immunity, M-cell development and mature have not been fully addressed.

Up to date, limited studies have been available about M-cell development. M cells differentiated from leucine-rich repeat-containing G-protein-coupled receptor 5^+^ (Lgr5^+^) stem cells and regulated by both the receptor activator of the nuclear factor-*κ*B ligand (RANKL) and transcription factor Spi-B [9–11]. Upon stimulation with RANKL, TNF receptor-associated factor 6 (TRAF6) activation triggered NF-*κ*B pathway. The canonical RelA/p50 activation led to induce expression of early M-cell (immature) markers such as Marcks like 1 (MarcksL1) and chemokine (C–C motif) ligand 9 (CCL9), CCL20, whereas noncanonical RelB/p52 activation caused Spi-B and Sox8 transcription factors expression, which was essential to maturation of M cells (marker: glycoprotein 2: GP2) [12–14]. Moreover, Spi-B depletion or conditional knockout TRAF6 [13], SRY-Box transcription factor 8 (SOX8) [12], or S100A4 [15] could lead to decreased M cells numbers *in vivo*, while a novel M-cell–specific transcription factor polycomb repressive complexes 2 (PRC2)-regulated estrogen-related–receptor g (Esrrg) has been demonstrated to promote M-cell development and differentiation [14]. In addition to these findings, signaling pathways required for M-cell differentiation and mature remained elusive.

Alteration of metabolic reprograming in IECs is associated with many diseases, which is one of the key etiological factors of inflammatory bowel disease (IBD) and other digestive tract diseases [16–18]. The polyol pathway, an alternative glucose metabolism, was believed to play an important role in explaining the pathogenesis of complications in patients with end-stage diabetes [19]. The work from Huang et al. [20] and Yang et al. [21], respectively, showed that aldose reductase (AR) involved in intestinal epithelial cell differentiation and AR-mediated sorbitol in plasmacytes triggered metalloprotease 2 (MMP2) to cleave peptidoglycan recognition receptor LC (PGRP-LC) in fat body to initiate systemic innate immune communication in drosophila [21, 22], suggesting the critical role of polyol pathway in intestinal epithelial cell differentiation and immunity. Herein, in this work, we further addressed sorbitol treatment caused a significant downregulation of early M-cell differentiation marker expression, including SOX8 and spi-B, and mature marker GP2 expression, leading to inhibit M-cell development, which was attributed to decreased RANKL expression *in vivo* and *in vitro*. Further results showed that sorbitol triggered phosphorylation of phosphodiesterase 4 (PDE4) in the M-cell model, leading to decreased protein kinase A (PKA)/cAMP-response element binding protein (CREB) signaling and nuclear translocation of CREB, which attenuated CREB binds to RANKL promoter, suppressing transcriptional activation of RANKL. What is more, inhibition of PDE4 could reverse the inhibitory effect of sorbitol on M-cell development *in vivo* and *in vitro*. Taken together, our study has extended the function of sorbitol and revealed a novel mechanism underlying M-cell development.

## 2. Materials and Methods

### 2.1. Reagents and Antibodies

Dulbecco's modified Eagle's medium/F12 (DMEM/F12) (GIBCO, C11330500BT) and fetal bovine serum (FBS) (GIBCO, 10099141C) were purchased from Life Technologies (Carlsbad, CA, USA); IntestiCult™ Organoid Growth Medium (mouse) (06005) and gentle cell dissociation reagent (07174) were purchased from STEMCELL Technologies, CryoStor CS10 (07930) was from Biolife Soultions. Corning® Matrigel® growth factor reduced (GFR) basement membrane matrix (356231) was from Corning. Phenylmethanesulfonyl fluoride (PMSF, P0100) and protease inhibitor cocktail (PIC, P6730) were from Solarbio (Beijing, China); Beyozol RNA Isolation Kit (R0011) was purchased from Beyotime Biotechnology (Shanghai, China); All-in-One First-Strand cDNA Synthesis Kit (QP006) and All-in-One quantitative polymerase chain reaction (qPCR) Mix (QP005) were obtained from GeneCopoeia (Rockville, MD, USA). D-Sorbitol (HY-B0400), D-Mannitol (HY-N0378), and Dipyridamole (DIP, HY-B0312) were from MedChemExpress (NJ, USA). RANKL monoclonal antibody (Proteintech, 66610-1-Ig); phospho-PDE4 (Immunoway, YP0668) was from Immunoway (Jiangsu, China), PKACA (Proteintech, 67491-1-Ig), CREB1 polyclonal antibody (Proteintech, 12208-1-AP); Phospho-CREB1 (Ser133) polyclonal antibody (Proteintech, 28792-1-AP), Lamin A/C polyclonal antibody (Proteintech, 10298-1-AP) and *α*-tubulin monoclonal antibody (Proteintech, 66031-1-Ig) were from Proteintech; phospho-PKA (Thr197) (CST, 5661) was from cell signaling technology (Danvers, MA, USA); peroxidase-affiniPure goat anti-rabbit IgG (H + L) (111-035-003) and peroxidase-affiniPure goat anti-mouse IgG (H + L) (115-035-003)were purchased from Jackson. Alexa-488- and 594-conjugated secondary antibodies were from Immunoway (Beijing, China).

### 2.2. Cell Lines, M-Cell Model, and Treatment

As described in a previous study [23], Raji B and CaCO_2_ cells were from the American Type Culture Collection and cultured in RPMI1640 and DMEM, respectively, supplemented with 10% FBS according to the manufacturer's recommendations. For the M-cell model, the CaCO_2_/Raji B coculture system was established as follows: 4 × 10^5^ CaCO_2_ cells were digested and seeded into the inserts in a 6-well plate, and 4 × 10^7^ Raji B cells were placed into the bottom of the insert. TEER was measured every 2 days, starting from day 0 to day 21, to monitor cell differentiation. The coculture was established for 4 days after CaCO_2_ cells was maintained for 3 weeks, and the medium changed every day. For treatment, mannitol and sorbitol were used at a final concentration of 100 mM; DIP was used at a final concentration of 6 *μ*M.

### 2.3. Crypt Isolation, Intestinal Organoid Culture, and Treatment

Small intestines were isolated from C57BL6 and immersed into ice DPBS (STEMCELL, 37350) to cut into 1–2 mm pieces, followed by suspension with GCDR (STEMCELL, 07174). Centrifugation at 290 *g* for 5 min was performed after incubation on a shaker at 20 rpm for 15 min at room temperature; the supernatant was removed after gravity settling for 30 s. The tissue fragment was resuspended with 10 mL cold phosphate buffer solution (PBS) containing 0.1% BSA and subjected by a 70 *μ*m cell strainer (Corning®, 352350) to collect the filtrate in a clean 50 mL tube to remove the tissue fragments. The crypts were resuspended with intestiCult™ medium (STEMCELL, 06005) containing 50% matrigel® (Corning®, 356231) after centrifugation at 200 *g* for 5 min at 4°C and seeded in the center of 24-plate (Corning®; 3526) to rest for 20 min until the Matrigel forms a dome, 750 *μ*L intestiCult™ medium were added into well to culture for 7–10 days in incubators at 37°C and 5% CO_2_ with changes of medium every 2–3 days. Enteroids were imaged daily under a microscope.

For treatment, the control group was treated with mannitol (100 mM) to rule out osmotic pressure and the sorbitol group was treated with sorbitol (100 mM), the treatment group was stimulated with sorbitol combined with 8 *μ*M RANKL or 6 *μ*M DIP. Medium was replaced every 2 days, and organoids were cultured for 7 days. On the 2nd, 4th, and 7th day of culture, we observed the effect of indicated treatments on the apparent changes of organoids during differentiation and maturation. On the 7th day of organoid culture, the supernatant was collected to measure RANKL expression in the supernatant. The total RNA level isolated from organoids was collected to detect relative gene expression, or the organoids were harvested to fix to prepare for immunofluorescence (IF) analysis.

### 2.4. RNA Extraction and qRT-PCR

As described in our previous study, total RNA from the indicated treatment was extracted and converted into cDNA according to the Beyozol RNA Isolation Kit and the All-in-One™ first-strand cDNA synthesis kit (Genecopoeia™, FulenGen), respectively. Quantitative PCR (qPCR) was carried out to detect gene expression using the All-in-One™ qPCR mix (Genecopoeia™, FulenGen) according to the manufacturer's instructions. Primer sequences used in this study were listed as followed: *GP2*: forward: 5′-AATGTGCGGGAGAATGGTGT-3′ and reverse: 5′-TCTGAGCACTGGTTGACACT-3′; *spib*: forward: 5′-ATCACAGCTGCCACCATCTC-3′ and reverse: 5′-ACAGCTTAAGTGTGGGCCAT-3′; *Marcksl1*: forward:5′-GGAGAATGGCCACGTGAGAA-3′ and reverse: 5′-TCGATGGCATCACCAGTAGC-3′; *SOX8*: forward: 5′-ATCATTGGGCCAGGCATTGA-3′ and reverse: 5′-GTTGGGGAGGCTCTCCTTTC-3′; *ANXA5*: forward: 5′-GACCGACAGCATCATGGCTA-3′ and reverse: 5′-AGCATTGCTTCGGGATGTCA-3′; *GAPDH*: forward: 5′-TGTGTCCGTCGTGGATCTG-3′ and reverse: 5′-CCTGCTTCACCACCTTCTTGA-3′.

### 2.5. IF

As described in the previous studies [24, 25], after dewaxing and dehydration, the slide of intestinal organoid was subjected from antigen retrieval and blocked with goat serum for 30 min. The slips were incubated with the primary antibody overnight at 4°C, followed by incubation with Alexa-488- or Alexa 594-conjugated secondary antibodies for 1 hr at room temperature. The coverslips were mounted onto glass slides with prolonged gold antifade reagent with DAPI, and stained cells were imaged under a laser scanning confocal fluorescent microscope.

### 2.6. Enzyme-Linked Immunosorbent Assay (ELISA)

RANKL level in the supernatant of the M-cell model in response to mannitol and sorbitol treatment was measured using Mouse RANKL ELISA Kit (Cloud Clone Corp. TX; SEA855Mu) according to manufactory instruction. Absorbances were measured at a wavelength of 450 nm, subtracting the values measured at 570 nm, using a microplate reader.

### 2.7. Western Blotting (WB) and Immunoprecipitation (IP)

As described in our work [20, 25], for IP, cells were lysed in ice-cold buffer composed of 50 mmol/L Tris–HCl (pH 7.4), 150 mmol/L NaCl, 0.1% NP-40 and protease inhibitors, followed by incubation overnight with anti-PKA, and further incubation was performed with protein A/G beads for 1 hr at 4°C. Beads were washed five times with low-salt lysis buffer, and immunoprecipitates were eluted for sodium dodecyl sulfate–polyacrylamide gel electrophoresis. For the immunoblotting, proteins were transferred into nitrocellulose (NC) membranes to incubate with primary antibodies overnight after blocking with 3% milk in PBST; the secondary antibodies were added to incubate for a further 1 hr at room temperature, and proteins were detected using an enhanced chemiluminescence (Perkin Elmer).

### 2.8. Luciferase Assay

As described in our work [25], the reporter plasmid containing RANKL promoter was transfected into HT-29 cells with internal control pGL4.74 for 24 hr, and mannitol and sorbitol were used to treat for another 24 hr, the relative luciferase unit was measured according to dual-luciferase reporter assay system (Promega).

### 2.9. Chromatin Immunoprecipitation (ChIP)

As described in previous studies [26, 27], CaCO_2_ cells were grown up to 80% confluence, the cell was treated with serum-free medium for 24 hr and stimulated with Mannitol or Sorbitol for another 1 hr, respectively, and the ChIP was performed according to the manufacturer's protocol to analyze the effect of sorbitol on CREB binds to RANKL promoter. Quantitative PCR of co-immunoprecipitated genomic DNA fragments was performed with specific primers was synthesized from thermolife.

### 2.10. Ethical Approval of Animal Studies

About 6–8 weeks, C57BL/6 mice were obtained from Southern Medical Univeristy to isolate crypt isolation for intestinal organoid experiments, which were housed in individually ventilated cages in a barrier facility proactive in environmental enrichment under specific pathogen-free conditions in line with European Union regulations. All experimental animal procedures were approved by the Institutional Animal Committee of Southern Medical University (SMUL2021156).

### 2.11. *In Vivo* Experiment

About 6–8 weeks, C57BL/6 mice were randomly grouped into three groups: mannitol group, sorbitol group, and sorbitol group received DIP treatment. In detail, mice received with mannitol or sorbitol alone dissolved in H_2_O at concertation of 100 mM for 1 week and combined with DIP for another 1 week. This 2-week cycle was repeated for 3 times. Mice were killed for intestine isolation to analyze M-cell differentiation.

### 2.12. Statistics Analysis

The data were displayed as mean ± s.e.m. and statistical analysis was conducted with GraphPad Prism nine software. The difference in qPCR assay was analyzed by one sample *t* test, and one-way ANOVA was used to determine the difference in RANKL level through ELISA assay. Two ANOVAs were used to analyze the difference in luciferase assays. A *p* value less than 0.05 was significant.

## 3. Results

### 3.1. Sorbitol-Inhibited M-Cell Development *In Vitro*

**Figure 1:**
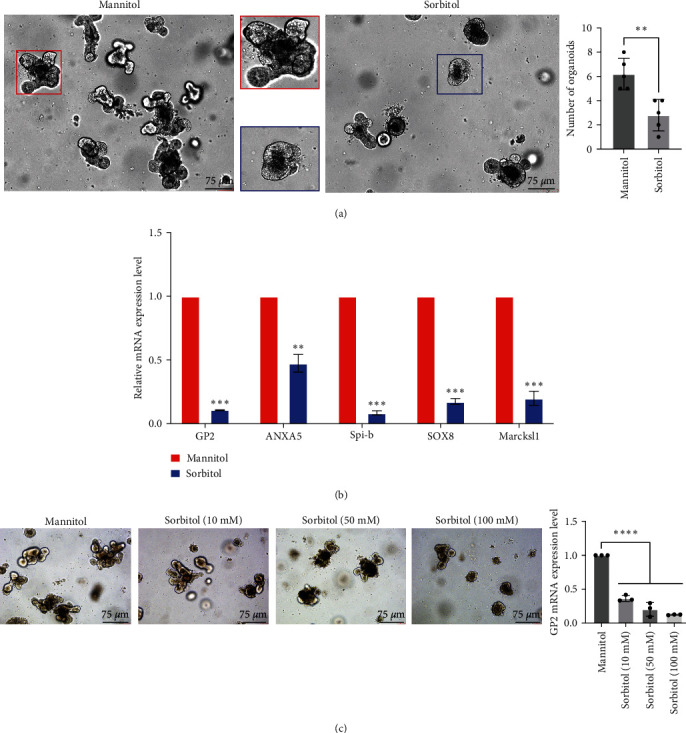
Sorbitol suppressed M-cell differentiation: (a) intestinal organoids were analyzed in response to 100 mM mannitol and 100 mM sorbitol. The representative image of intestinal organoids was captured to analyze the effect of sorbitol on M-cell differentiation; (b) the total RNA was extracted from the indicated group described in (a), and real-time PCR was employed to detect related gene expression involved in M-cell differentiation, including GP2, ANXA5, spi-B, SOX8, and Marckl1. Data presented as the mean ± s.e.m. of three independent experiments and were analyzed by one sample *t* test,  ^*∗∗∗*^*p* < 0.001,  ^*∗∗*^*p* < 0.01; (c) the representative image of intestinal organoids was imaged in response to various concertation of sorbitol. Bar: 75 *μ*m, data presented as the mean ± s.e.m. were analyzed by one ANOVA,  ^*∗∗∗∗*^*p* < 0.0001.

To further determine the potential role of sorbitol on M-cell development, intestinal organoid combined with IF and qPCR was used to identify the relative gene expression changes involved in the differentiation and maturity of M cells. As shown in Figure 1(a), mannitol was employed as a control group to rule out osmotic pressure, and sorbitol treatment led to a significantly impaired intestinal organoid. qPCR results from intestinal organoid further showed that in comparison with the mannitol group, downregulation of genes involved in M-cell differentiation, including ANXA5, Spi-B, SOX8, and Marcksl1 were obviously observed in response to sorbitol stimulation. In addition, Mature M cells marker GP2 was largely inhibited by sorbitol (Figure 1(b)). Further analysis showed that impaired intestinal organoid was observed in response to sorbitol stimulation in a dose-dependent manner (Figure 1(c)). What is more, the apoptosis-related protein expressions, including caspase3 and Bax, were increased, while BCL-2 and ki67 expressions were decreased in response to sorbitol treatment (Figure S1(a)); in addition, caspase3 mRNA level expression was increased in response to sorbitol treatment (Figure S1(b)). These findings suggested that sorbitol inhibited the development of intestinal M cells.

### 3.2. Sorbitol-Suppressed M-Cell Differentiation through RANKL

**Figure 2:**
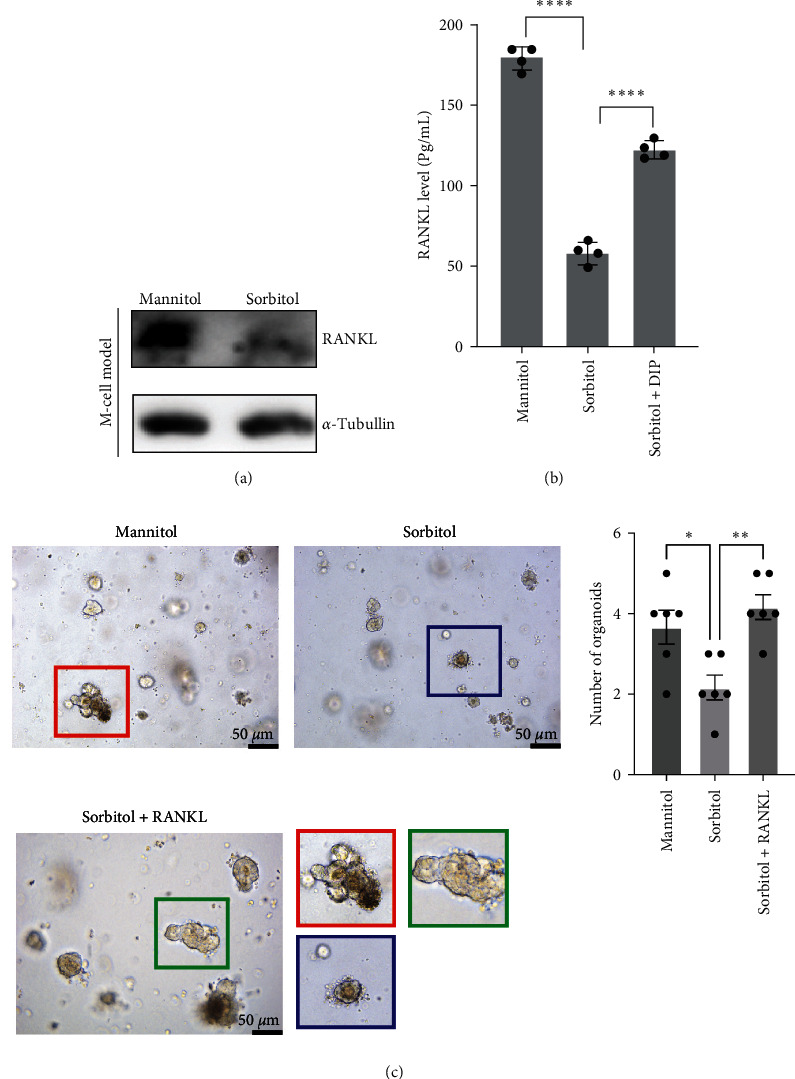
Sorbitol regulated M-cell differentiation through PDE4/PKA/CREB signaling-mediated RANKL: (a) after establishing M-cell model, the total protein was extracted from M-cell model treated with 100 mM mannitol or 100 mM sorbitol for 24 hr, western blotting was used to detect RANKL; (b) the M cells was established and treated described in (a), and the concentration of RANKL level in supernatant was measured in indicated group through ELISA assay. Data presented as the mean ± s.e.m. of three independent experiments and were analyzed by *t* test,  ^*∗∗∗∗*^*p* < 0.001; (c) intestinal organoids culture was performed to visualize the effect of PDE4 inhibition by DIP on sorbitol-mediated M-cell development. Bar: 50 *μ*m. Data presented as the mean ± s.e.m. and were analyzed by one ANOVA,  ^*∗∗*^*p* < 0.01,  ^*∗*^*p* < 0.05.

The above results implied that sorbitol played an important role in regulating intestinal M-cell development. RANKL has been reported to induce mature marker GP2 expression during M-cell differentiation [28, 29], which focused us to explore whether sorbitol-regulated M-cell development is dependent on RANKL. As shown in Figures 2(a) and 2(b), in comparison with mannitol, both the results from WB and ELISA have shown that RANKL was largely decreased in intestinal organoid treated with sorbitol at the protein level in the M-cell model. What's more, the addition of recombinant of RANKL could reverse the impaired effect of sorbitol on intestinal organoid (Figure 2(c)). Taken together, these results suggested sorbitol destroyed intestinal organoids through RANKL.

### 3.3. CREB was Required for Sorbitol-Mediated RANKL Expression

**Figure 3:**
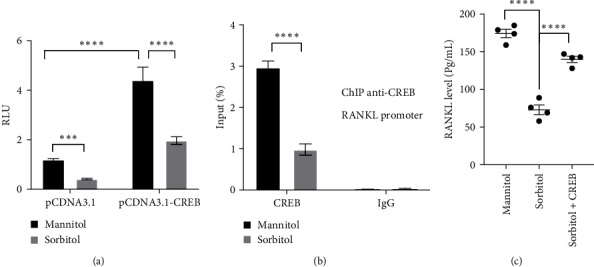
CREB is critical for sorbitol-mediated RANKL expression: (a) reporter gene containing RANKL promoter was transfected into CaCO_2_ cells combined with pGL4.74 plasmid for 24 hr, CaCO_2_ cell was treated with 100 mM mannitol and 100 mM sorbitol for further 24 hr, the relative luciferase unit was detected using dual-luciferase reporter assay system. Data presented as the mean ± s.e.m. of three independent experiments and were analyzed by two ANOVA,  ^*∗∗∗*^*p* < 0.001,  ^*∗∗∗∗*^*p* < 0.0001; (b) after serum-free for 24 hr, CaCO_2_ cells were stimulated for 1 hr, and the whole cell was fixed and lysed to incubate with an anti-CREB antibody, the chromosome fragments binding to CREB were amplified and quantified by real-time PCR with RANKL promoter primer. Data presented as the mean ± s.e.m. of three independent experiments and were analyzed by two ANOVA,  ^*∗∗∗∗*^*p* < 0.0001; (c) ELISA was employed to detect RANKL level in indicated group in CaCO_2_ cells, data presented as the mean ± s.e.m. of five independent experiments and were analyzed by one ANOVA,  ^*∗∗∗∗*^*p* < 0.0001.

The binding of CREB to the RANKL promoter and subsequent transcription activation has been addressed in a large number of studies [30, 31], which focused us to seek whether sorbitol-regulated RANKL expression is dependent on CREB. As shown in Figure 3(a), overexpression of CREB significantly enhanced the relative luminescence unit (RLU) of RANKL in HT-29 cells transfected with a reporter gene containing RANKL promoter and pGL4.74 in the mannitol group, while sorbitol treatment largely blocked the promotion of CREB on RANKL luminescence. Moreover, ChIP revealed that in comparison with mannitol, sorbitol stimulation led to a significant downregulation binding of CREB to the RANKL promoter (Figure 3(b)). What is more, ectopic expression of CREB in CaCO_2_ cells largely rescued the inhibitory effect of sorbitol on RANKL expression and secretion (Figure 3(c)). These results suggested that CREB is required for sorbitol-derived RANKL expression.

### 3.4. Sorbitol-Modulated PDE4/PKA/CREB Cascade Signaling

**Figure 4:**
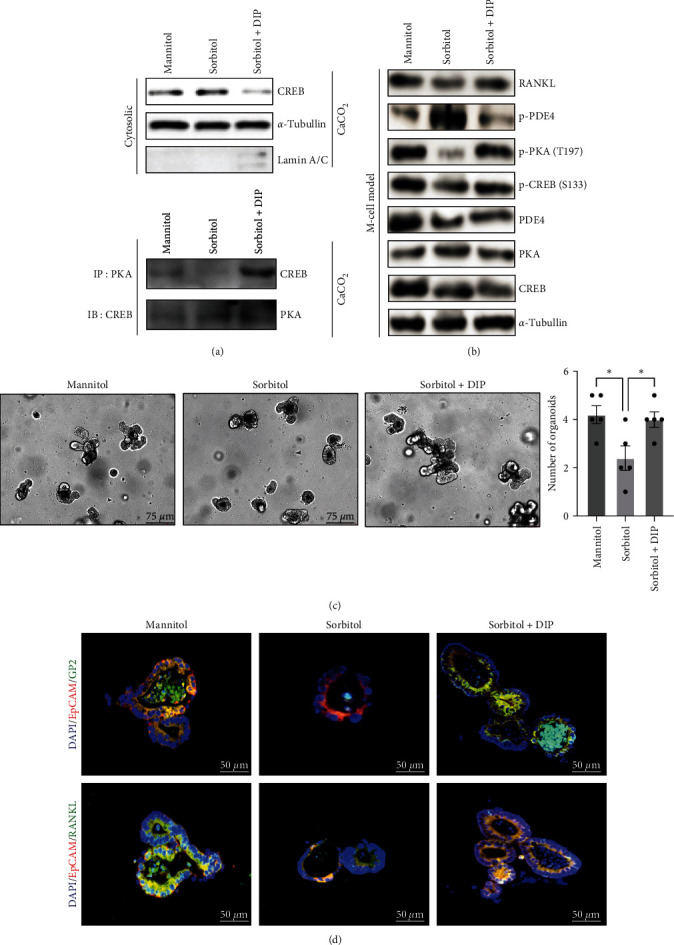
Sorbitol modulated PDE4/PKA/CREB signaling: (a) *upper panel:* CaCO_2_ cells were serum starved for 24 hr and stimulated with mannitol, sorbitol, and sorbitol+DIP for further 1 hr. Cell fraction was isolated and levels of cytosolic CREB were detected by western blotting. *α*-Tubulin and lamin A/C were used as internal controls for the cytosolic and nuclear fractions, respectively. *Bottom panel:* CaCO_2_ cells were confluence, serum starved for 24 hr, then stimulated with 100 mM mannitol or sorbitol combined with or without DIP for 1 hr. Immunoprecipitation was performed with antibodies targeting endogenous PKA and immunoblotting was used to detect CREB; (b) after establishing M-cell model, the total protein was extracted from M-cell model treated with 100 mM mannitol or 100 mM sorbitol and 100 mM sorbitol + DIP for 24 hr, western blotting was used to detect RANKL, baseline and phosphorylation of PDE4/PKA/CREB. *α*-tubulin was served as an internal control; (c) intestinal organoids culture was performed to analyze the mannitol, sorbitol, sorbitol + DIP on differentiation. Data presented as the mean ± s.e.m. of five independent experiments and were analyzed by one ANOVA,  ^*∗*^*p* < 0.05; (d) IF was used to detect M cells mature marker GP2 expression and RANKL expression in the indicated group.

CREB is the critical transcript factor in cAMP signaling; targeting to inhibit PDE4 by dipyridamole (DIP) could enrich CD8^+^CD39^+^T cells abundance and enhance CDX2 in IECs, alleviating intestinal inflammation and inducing mucosa healing [32, 33]. The M-cell model was used to analyze the potential changes of PDE4/PKA/CREB signaling in response to sorbitol stimulation [34, 35]. The results from WB demonstrated that sorbitol treatment led to a significant retention of cytosolic CREB, which was attributed to impaired interaction between PKA and CREB caused by sorbitol (Figure 4(a)). The work further showed that sorbitol triggered activation of PDE4, leading to inhibited phosphorylation of PKA (Thr197) and CREB (Ser133) confirmed by the immunoblotting from the M-cell model, despite no significant difference was observed in the baseline of PDE4/PKA/CREB (Figure 2(b)). What is more, inhibition of PDE4 by DIP largely blocked the effect of sorbitol on PDE4/PKA/CREB signaling, leading to increased RANKL secretion (Figure 4(b)). Most importantly, intestinal organoids have further confirmed that inhibition of PDE4 by DIP could reverse the sorbitol on intestinal organoids development, leading to enhanced GP2 and RANKL expression (Figures 4(c) and 4(d)). Taken together, these findings suggested that sorbitol suppressed RANKL expression through modulating PDE4/PKA/CREB signaling.

### 3.5. DIP Improved Sorbitol-Mediated M-Cell Differentiation Inhibition *In Vivo*

**Figure 5:**
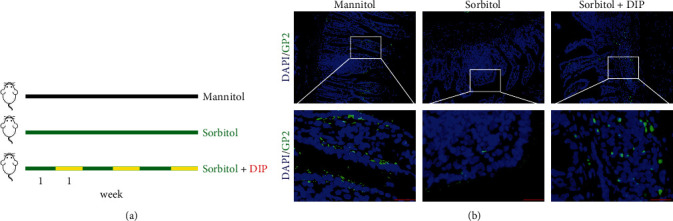
PDE4 inhibition rescued sorbitol-mediated M-cell development: (a) a schematic model of mice received mannitol or sorbitol or sorbitol combined with DIP (6 *μ*M) for 6 weeks to establish a chronic model; (b) IF analysis of mature M cells labeled with GP2 in the intestine from the indicated group. Bar: 75 *μ*m.

To confirm whether inhibition of PDE4 could rescue the effect of sorbitol on M-cell differentiation *in vivo*. The *in vivo* model was established as described in Yang et al. [36] work with brief modification. As shown in Figure 5(a), mice were fed with water supplemented with 2% (wt/vol) mannitol or sorbitol for 7 days and combined with PDE4 inhibitor DIP treatment for another week. This 2-week treatment was repeated for three cycles. On the last day, the intestine was isolated for analysis of M cells after mice were euthanized. As expected, the results showed that, in comparison with mannitol group, sorbitol treatment led to a significant inhibition of mature M cells labeled with GP2, while DIP treatment reversed the inhibitory effect of sorbitol on M cells development (Figure 5(b)). Collectively, this work suggested that targeting PDE4 by DIP could be a promising strategy to induce M-cell differentiation.

## 4. Discussion

Metabolites or metabolic reprograming is critical for cell fate. Up to now, to our best knowledge, there are no available reports about the function of metabolites or metabolic reprograming on intestinal M-cell development. In this work, we demonstrated sorbitol, the production of the polyol pathway, and derived intestinal organoid damage through the reduction of RANKL expression. Mechanically, activation of PDE4 was obviously observed in the M-cell model after treatment with sorbitol, which further led to inhibited phosphorylation of PKA/CREB, reducing CREB nuclear translocation and decreasing the binding of CREB to RANKL promoter. What is more, endogenous PKA interacted with CREB, and this interaction was disrupted by sorbitol stimulation. Most importantly, inhibition of PDE4 by DIP could overcome the inhibitory effect of sorbitol on intestinal M-cell development *in vivo* and *in vitro*. These findings extended the role of sorbitol in M-cell differentiation and suggested targeting to inhibit PDE4 by dipyridamole, which is a promising strategy for intestinal M-cell development.

The polyol pathway, the conversion of glucose into sorbitol, is almost silent, which is activated in hyperglycemic conditions and has deleterious effects on human health [19, 37]. In addition, the polyol pathway is necessary for ChREBP nuclear localization in hepatocytes and glucose tolerance in mice, and long-term uptake of sorbitol could induce a significant change in the composition of the gut microbiome [38]. The further work showed that sorbitol could induce an upregulation of Aquaporins 7 expression in a time-dependent manner [39] and apoptosis [40, 41]. What is more, sorbitol was found to be able to relay gut-fat body immunological communication (GFIC) by activation of metalloprotease 2, which further cleaved PGRP-LC to activate immune deficiency response in fat bodies [21]. In this work, we further extended the novel role of sorbitol in gastroenterology that sorbitol suppressed intestinal M-cell differentiation and matured through RANK *in vivo* and *in vitro*, which was attributed to the activation of PDE4. Inhibition of PDE4B by DIP could rescue the inhibitory effect of sorbitol on M-cell development. However, in addition to M cells, the further work is required to address the potential function of sorbitol in other intestinal epithelial cell development, including goblet cells, paneth cells, and tuft cells.

The classical second messenger cAMP pathway has been confirmed to be essential for a variety of physiological functions, including mitochondrial biology, lipid metabolism, ischemia, and inflammation [42]. Recent work has demonstrated that inhibition of PDE4 by apremilast modulated cAMP-predominant PKA-CREB signaling ameliorated the clinical symptoms of chronic UC as evidenced by improvement in mucosal ulcerations, tissue fibrosis, and inflammatory infiltrations [43, 44]. Our previous work has suggested that PDE4 inhibition could lead to CDX2 expression, leading to intestinal epithelial cell differentiation [33]. In this work, inhibition of PDE4 could alleviate sorbitol-induced impaired M-cell development. These works suggested that PDE4 activity is critical for M-cell development. However, the further work was required to address how sorbitol activated PDE4 phosphorylation, the receptor of sorbitol is urgently to be identified, and whether the sorbitol receptor involved in M-cell development remained elusive.

Taken together, these findings extended the role of sorbitol and established the mechanism through which sorbitol regulated M-cell development by activation of PDE4, which could be a theoretical foundation for DIP used in maintaining mucosal immunity function.

## Data Availability

The datasets generated during and/or analyses during the current study are available from the corresponding author upon reasonable request.

## Abbreviations

ANXA5: Annexin 5
AR: Aldose reductase
AQP7: Aquaporins 7
CREB: cAMP-response element-binding protein
CCL: Chemokine (C–C motif) ligand
ChIP: Chromatin immunoprecipitation
DMEM: Dulbecco's modified Eagle medium
DIP: Dipyridamole
DMEM: Dulbecco's modified Eagle's medium/F12
Esrrg: Estrogen-related receptor g
FBS: Fetal bovine serum
GP2: Glycoprotein 2
GFIC: Gut-fat body immunological communication
IF: Immunofluorescence
IBD: Inflammatory bowel disease
IECs: Intestinal epithelial cells
Lgr5: Leucine-rich repeat-containing G-protein-coupled receptor 5
M cells: Microfold cells
MMP2: Metalloprotease 2
MARCKSL1: MARCKS-like protein 1
PRC2: Polycomb repressive complexes 2
PKA: Protein kinase A
PDE4: Phosphodiesterase 4
PBS: Phosphate Buffer solution
PMSF: Phenylmethanesulfonyl fluoride
PGRP-LC: Peptidoglycan recognition receptor LC
qPCR: Quantitative polymerase chain reaction
RANKL: Receptor activator of nuclear factor kappa-В ligand
SOX8: SRY-Box transcription factor 8
SDS–PAGE: Sodium dodecyl sulfate–polyacrylamide gel electrophoresis
TRAF6: TNF receptor-associated factor 6
TBST: Tris-buffered saline tween-20
WB: Western blotting.

## Supplementary Materials

Supplementary Materials Figure S1: the effect of sorbitol on M-cell apoptosis.
