# Supplementary figures and images for: Sorbitol Destroyed Intestinal Microfold Cells (M Cells) Development through Inhibition of PDE4-Mediated RANKL Expression
Source: Mediators Inflamm. 2024 May 2;2024:7524314. doi: 10.1155/2024/7524314 (PMC11081746; doi:10.1155/2024/7524314)

**Fig.S1**

**A**

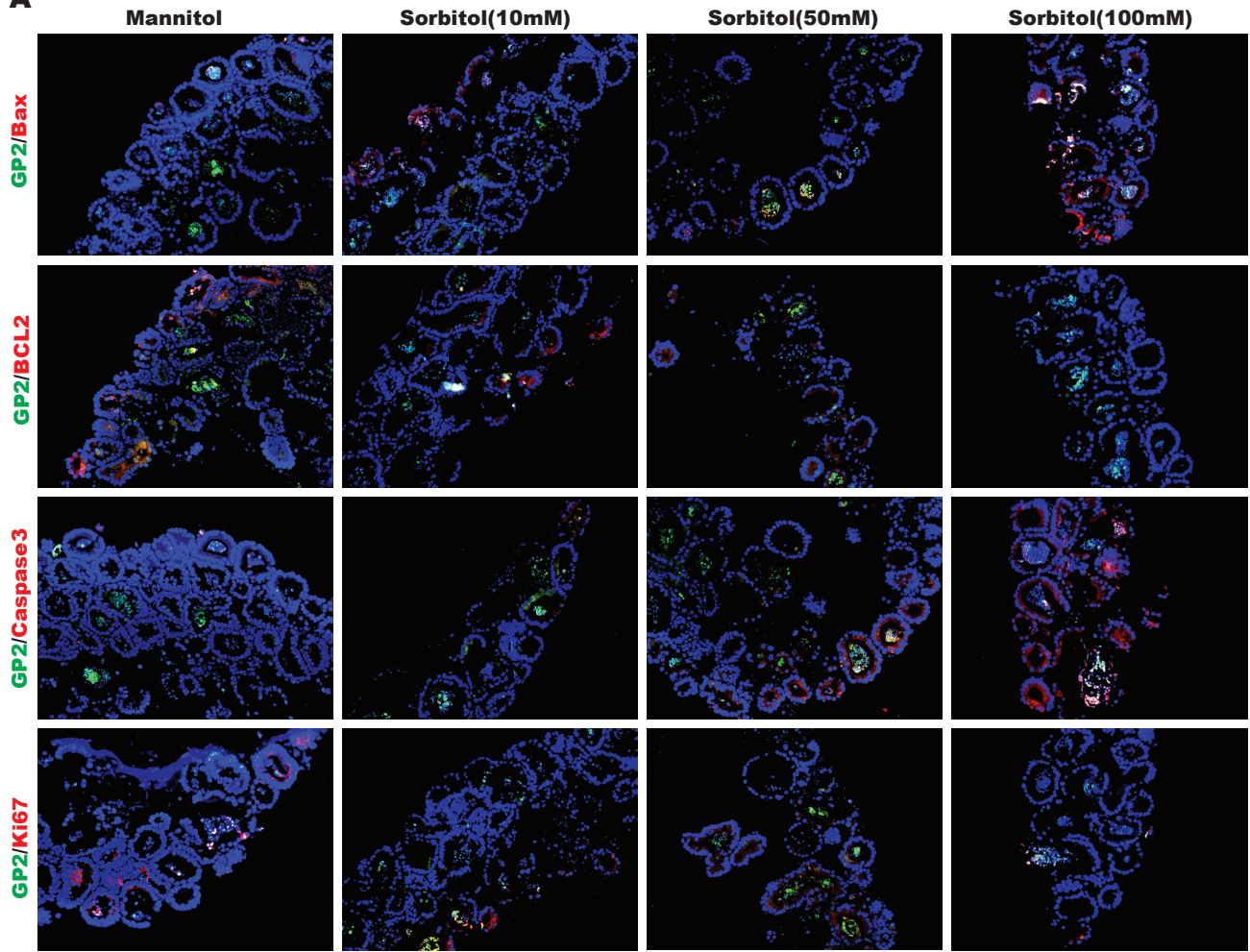

**B**

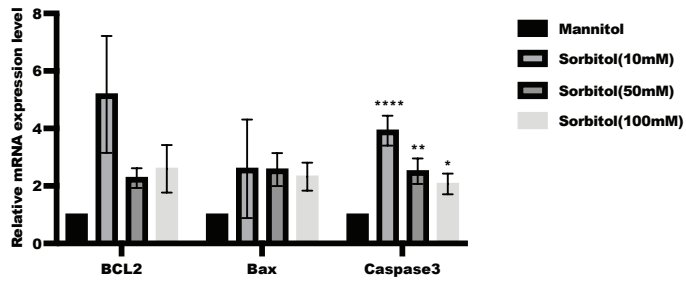

Supplement: Supplementary Materials — Figure S1: the effect of sorbitol on M-cell apoptosis. [file 7524314.f1.pdf]
